# Estimating the Economic Impact and Assessing Owners' Knowledge and Practices of Epizootic Lymphangitis in Equine Cart Animals in Central and South Gondar Zones, Amhara Region, Ethiopia

**DOI:** 10.3389/fvets.2021.673442

**Published:** 2021-06-16

**Authors:** Amsalu Misgie Molla, Tewodros Fentahun, Wudu T. Jemberu

**Affiliations:** ^1^Department of Animal Sciences, College of Agriculture and Natural Resources, Mekidela Amba University, Tulu Awulia, Ethiopia; ^2^Department of Biomedical Sciences, University of Gondar, Gondar, Ethiopia; ^3^Department of Veterinary Epidemiology and Public Health, University of Gondar, Gondar, Ethiopia

**Keywords:** cart animals, economic impact, epizootic lymphangitis, equids, Ethiopia, knowledge, practice

## Abstract

Epizootic lymphangitis (EL) is a chronic, contagious, fungal disease of equids. The disease is highly prevalent in cart pulling equines of Ethiopia affecting the livelihood of the cart owning households and welfare of the cart animals. This study estimated the economic impacts of EL and assessed cart owners' knowledge and practices related to the disease in northwest Ethiopia. A multistage cluster sampling approach was implemented to select cart animal owners for the study. A total of 274 cart animal owners were interviewed to collect data for the study. The average annual economic loss per cart owner was estimated at Ethiopian Birr (ETB) 8447. Of this loss, the ETB 4364, 2838, and 1245 were due to mortality, working power loss, and treatment costs, respectively. When the loss was computed only for affected car owners, it was on average ETB 9835 per affected cart owner. The average annual animal level loss was estimated at ETB 6587 per cart animal. Mortality was the largest contributor of the overall economic losses. There was a statistically significant difference in average economic losses per household between study towns (*P* < 0.05). The knowledge and practice study revealed that 51.2% of the respondents had good knowledge level (knowledge score above the mean score) of EL, but only 45.2% of the respondents had a good practice related to EL. A multivariable logistic regression analysis revealed that socio-demographic factors such as educational level and town of residence were significantly associated with EL knowledge level of the respondents (*P* < 0.05), and on the other hand, knowledge level and residence of the respondents were significantly associated with EL practice level (*P* < 0.05). The study generally indicates that EL causes significant economic impact on the cart business, but cart owners had insufficient knowledge and poor control and preventive practices to combat the disease. Attention should be accorded to control the disease and reduce its impact on the livelihood of cart owners. As part of the control measure, more awareness creation about the disease and its preventive and management measures for cart owners will very important.

## Introduction

Working equids play important role in Ethiopia's agricultural and transport systems. They are the main means of transportation of agricultural inputs and outputs in the dominant subsistence agricultural activities of the country (1). Equines are also used in the transport of people, firewood, water, construction, and waste materials (2, 3). Horse and mule drawn carts are vital transport means in most towns, and cart business is a source of livelihood for significant proportion of urban population of Ethiopia (4–6).

Epizootic lymphangitis (EL) is a debilitating contagious disease of working equids, caused by the dimorphic fungus *Histoplasma capsulatum* var. *farciminosum*, which is transmitted through contact of infected material with traumatized skin, by biting flies and ticks, or inhalation of spores (7, 8). The disease occurs clinically as cutaneous, ocular, respiratory, or mixed clinical forms, and the cutaneous form is the most common (7, 9). EL is currently endemic in the regions of Sub-Saharan Africa (8, 10). The disease is highly prevalent in Ethiopia; depending on the region, the prevalence of EL in cart animals varies from 0 to 44% (11–14).

The high prevalence of EL in Ethiopia poses a great threat to the cart business (6, 11). The use of horse or mule drawn carts to generate income is a means of survival for a significant number of Ethiopian households and provides an affordable transportation service in many towns (11). EL causes significant damage to the incomes of cart animal owners due to lesser pulling/loading capacity of the diseased animals, unwillingness of customers to use carts pulled by infected animals, treatment costs, and absence from work and death of affected cart animals (4–6). EL is also a serious animal welfare problem. Due to chronic nature of the disease and unavailability of effective treatment, affected horses and mules are often made to work until they are severely debilitated by the disease and unable to work and generate income. Finally, at advanced stage of the disease, they are abandoned and die with their welfare gravely compromised (5, 6). Generally, the impact of EL is multi-dimensional and encompasses effects upon the cart animal, the livelihoods of individual owner or driver, and the wider society (6, 15, 16).

Quantitative information on the economic impact of EL is essential to comprehend the magnitude of the effect and to develop effective disease control and prevention strategies. However, there is scant information on the estimate of economic losses associated with the disease except two studies in central Ethiopia (4, 6), which indicated that losses to the owner due to morbidity of a horse with EL can result to up to 50% reduction in daily earnings. Epidemiological studies in Amhara National Regional State showed that EL is a serious problem in cart horses and mules in several towns of the region, with a prevalence ranging from 15 to 23% (11–13, 17), but no study was available that shows the quantitative estimates of the economic impact of disease in the region. The current study is aimed at quantifying the economic impacts of EL on cart animal owners' livelihood and assessing the owners' knowledge and practices related to the disease in Amhara region, northwest Ethiopia.

## Materials and Methods

### Study Area, Population, and Design

**Figure 1:**
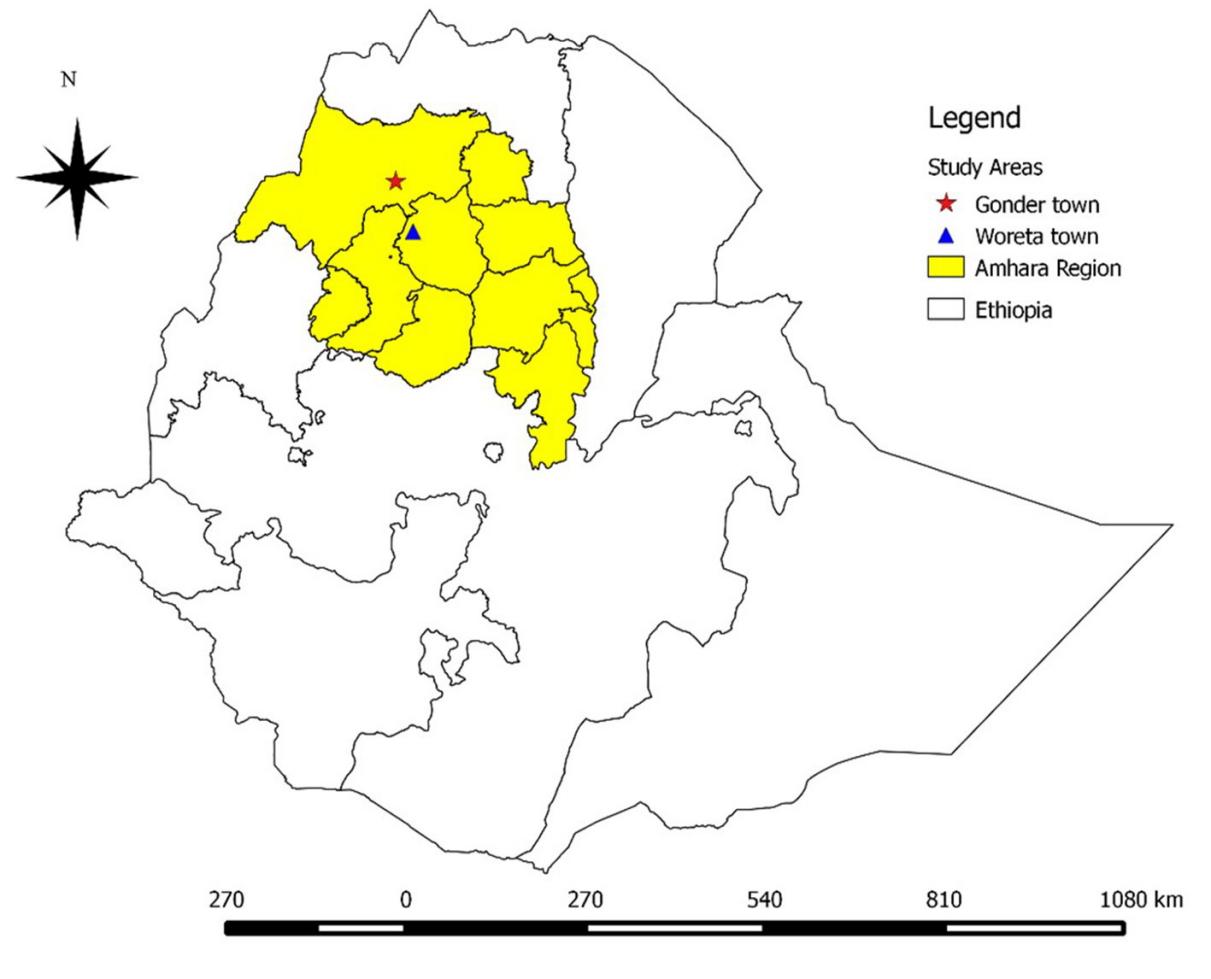
Map of study areas.

The study was conducted in two selected towns (Gondar town from Central Gondar zone and Woreta town from South Gondar zone) of Amhara regional state (Figure 1) during the period from December 2018 to May 2019.

Gondar town is found in Central Gondar zone. The town is located at 12°45′ North latitude and 37°45′ East longitude. It has an average elevation of 2,133 m above sea level (m.a.s.l.), average annual temperature of 19.3°C, and mean annual rainfall of 1, 200 mm (18). The cart horse population in the town is about 1,100 (19). Woreta town is found in the South Gondar zone located at 11°55′ North latitude and 37°42 East longitude. It has an elevation of 1, 828 m.a.s.l. with an average annual temperature of 20.3°C and mean annual rainfall of 1216.3 mm. The town has about a cart mule population of 313 (20).

The study population was cart horses and mules owners (hereafter cart owners) in the study towns. Cart horses and mules are those horses and mules that are used for cart pulling in transportation of humans and goods. In Gondar town, only horses and, in Woreta town, only mules were used for cart pulling purpose.

The study was based on a cross-sectional questionnaire survey, which was administered to cart owners to assess the economic impact of the disease, and knowledge and practices related to EL. The questionnaire was administered by face-to-face interview to cart owners. Before the interview, oral consent was obtained from each participant cart owner after detailed explanation on the purpose of the study, the risks and benefits of participation in the study, the right to refuse to participate in the study, as well as the conditions of confidentiality regarding the presentation of answers.

### Sampling Strategies

A multistage cluster sampling approach was implemented to select the respondent cart owners, in which town in the study zones was the primary sampling units and individual cart owners were the secondary sampling units. At stage one, two towns in the two zones (Gondar town from Central Gondar zone and Woreta town from South Gondar zone) were selected purposively based upon previous EL prevalence reports and confirmation of the active cases identified by a pilot survey. In stage two, individual cart owners from each town were selected using a simple random sampling technique. The lists of cart owners of those selected towns were obtained from their respective municipality and/or cart association's offices.

The randomly selected cart owners in those towns were used for both economic impact and knowledge and practice assessment components of the study. The selected cart owners were approached at cart horse and mule gathering sites such as markets, veterinary clinics, and cart stations. For those selected cart owners who were not met in these sites, a home visit was made.

### Sample Size Determination

The sample size for economic impact and the knowledge and practice assessment study was determined by using the sample size for estimating proportion formula provided in Thrusfield (21) (Formula 1). A previous study in the area reported about 23.2% prevalence of the disease (13), and assuming that owners with an affected horse or mule know about the disease and its impact, a sample size for estimation of proportion using expected proportion of 23.2%, 95% confidence level, and 5% required absolute precision was used to determine the sample size.

(1) $$\begin{array}{l} \text{n}={\left(1.96\right)}^{2}\left[\text{Pexp}\left(1-\text{Pexp}\right)\right]/{\text{d}}^{2} \end{array}$$

where *n* is the required sample size, Pexp is the expected proportion, and d is the desired absolute precision.

Accordingly, a sample size of 274 cart owner respondents was determined for the study. The sample size was distributed among the two study towns proportional to the cart owner population, resulting in 226 cart owners from Gondar town and 48 cart owners from Woreta town. Among 274 cart owners, 26 (8 from Gondar town and 18 from Woreta town) of them were not aware of EL and unable to describe the clinical signs associated with the disease, so they were not able to continue with the questionnaire. As a result, only 248 of the randomly selected cart animal owners were used in the economic impact and knowledge and practice assessments.

### Data Collection Methods

#### Questionnaire Survey

Data for the assessment of economic impact of the disease and knowledge and practice related to EL was collected using a structured questionnaire (Supplementary Material 1). The questionnaire had two parts: part one was designed primarily to assess and record data on economic impact parameters (morbidity and mortality, treatment cost, and reduction in work power due to illness), and part two was designed to assess the knowledge and practices of the respondents related to EL.

The questionnaire was administered by face-to-face interview. At the beginning of the interview, the respondents were asked whether they knew the disease by mentioning the local name of the disease “Nidift.” If they claimed they knew it, they were asked to mention the main clinical symptoms of the disease. If they mentioned the minimum set of clinical symptoms described in the case definition predefined in the questionnaire, they were considered as they knew the disease and continued with the interview and dropped from the interview if otherwise.

### Estimation of Economic Impacts

The economic impact of EL in cart horses and mules was determined according to the framework of Rushton (22), which classified economic impact of animal diseases in an endemic situation as direct and indirect impacts. Thus, quantifications of economic impact of EL had considered an estimation of the direct visible losses such as mortality loss, working power loss, and indirect impacts such as treatment cost and extra labor cost. The economic losses were estimated per cart owner per year, per cart animal per year, and per EL affected cart owners per year (cart owners who encountered EL in their cart horse or mule). Data for estimation of these impacts was derived from the responses of the individual cart animal owners to the economic impact assessment questionnaire. The economic impact estimation were done for a period of 1 year prior to the date of interview. All monetary impacts were recorded in Ethiopian Birr (ETB), which had mean exchange rate of 1 USD to 27.67 ETB for the study year 2018.

#### Mortality Loss

The mortality loss was set equal to the price of the animal that died if it were sold in the market while alive. Thus, the economic loss due to mortality per individual cart owner was calculated by considering the number of cart animals that died and their corresponding local market price (Formula 2).

(2) $$\begin{array}{l} {\text{MEL}}_{\text{i}}={\text{NM}}_{\text{i}}\;*\text{ P} \end{array}$$

where MEL_i_ represents the annual economic losses due to EL induced death of cart animal in cart owner i, NM_i_ is the number of cart animals that died in owner i, and P is the average price of the dead animals in the study year.

#### Working Power Loss

EL affected cart horse and mules cannot work to the same capacity that they can when in a healthy state. EL affected equids typically need to work fewer hours or a shorter day than healthy equids, and are limited to covering shorter distances and carrying lighter loads than healthy equids. For this study, the reduction in working power was captured as reduced effective working hours per day (which later changed into days) during illness period (Formula 3).

(3) $$\begin{array}{l} {\text{Lwork}}_{\text{i}}={\text{Ncart}}_{\text{i}}\;*\;\left(\text{Twor}{\text{k}}_{\text{i}}\;*\text{ adj}\right)\;*\text{ Prent} \end{array}$$

where Lwork_i_ represents the economic loss due to working power loss of the cart animal in cart owner i, Ncart_i_ is the number of cart horses or mules affected per year in owner i, Twork_i_ is the average duration of illness in days of affected horse or mule without work in owner i, adj is an adjustment factor for effective working days, and Prent is the price of daily rent of cart animal. According to the information from the owners, a cart animal has a break of 1 day in a week, which means 4 days in a month (around 50 days in a year) and about 10 public holiday days, totally 60 non-working days in a year. The probability that a day on which a cart animal is ill coincides with an effective working day was estimated as 305/365 (0.83). This ratio was used as an adjustment factor (adj) to change the days of illness to actual effective working days lost.

#### Treatment Costs

The economic cost of EL treatment per individual cart animal owners was calculated by considering medication costs and extra labor costs for seeking treatment for sick animals as given by Formula (4).

(4) $$\begin{array}{l} {\text{TrCost}}_{\text{i}}=\left({\text{NTr}}_{\text{i}}\;*\;{\text{PTr}}_{\text{i}}\right)+\left({\text{NhoursL}}_{\text{i}}\;*\;{\text{Pdl}}_{\text{i}}\right) \end{array}$$

where TrCost_i_ represents the treatment cost for affected cart animal in owner i, NTr_i_ is the number of animals treated, PTr_i_ is the average per head expenditure to EL treatment, NhoursL_i_ is the average number of working hours lost for nursing and seeking treatment for sick animals, and Pdl_i_ is the average payment rate of a replacement labor per hour.

#### Overall Economic Losses

The total annual economic losses (TEL) due to the occurrence of EL per individual cart animal owner were obtained by adding all the losses arising from mortality loss, working power loss, and treatment costs as given by Formula (5).

(5) $$\begin{array}{l} {\text{TEL}}_{\text{i}}={\text{MEL}}_{\text{i}}+{\text{Lwork}}_{\text{i}}+{\text{TrCost}}_{\text{i}} \end{array}$$

where TEL_i_ represents the total economic losses in cart animal owner i, MEL_i_ represents the economic losses due to EL induced death in cart animal owner i, Lwork_i_ represents the economic loss due to working power loss, and TrCost_i_ represents the treatment cost for EL affected in cart owner i.

### Determining Knowledge and Practice Level of Cart Animal Owners

A total of 10 knowledge questions that were scored out of a maximum obtainable score of 16 were administered to the cart owners. Knowledge scores for all respondents were normally distributed (Shapiro–Wilk test; *P* = 0.693). For normally distributed data (in our case knowledge score), mean is a good central value (23). Respondents mean knowledge score was taken as a cut off point for the knowledge level. Knowledge level of the respondents was categorized as “good” if the knowledge score was greater or equal to mean score and as “poor” if the score was less than the mean score.

A total of 11 practice questions that were scored out of a maximum obtainable score of 11 were administered to the cart owners. Practice score was generated for all respondents and was found to be non-normally distributed (Shapiro–Wilk test; *P* = 0.01). For data not normally distributed (in our case practice score), median is a good central value (23). Respondents' median practice score was taken as a cut off point for practice level. Practice level of the respondents was categorized as “good” if the score was greater than or equal to the median score and as “poor” if the score was less than the median score.

### Data Management and Statistical Analysis

All the data collected were entered and managed in Microsoft Excel (Microsoft Excel 2013, Microsoft Corporation, USA). STATA version 14 (Stata Corp., College Station, TX, USA) statistical analysis software was used to analyze the data. Descriptive statistics (frequency, percentage, average, and tables) were used to describe and summerize the data.

An independent sample *t*-test was utilized to evaluate differences in the average annual economic losses between towns. Multivariable logistic regression was utilized to identify socio-demographic factors associated with respondents' EL knowledge and practice levels. The influence of knowledge on the level of practices against EL was also evaluated using logistic regression. In all the analyses, the confidence level was held at 95% and *P* < 0.05 was set for statistical significance.

## Results

### Economic Impacts

The annual average economic losses computed from mortality loss, working power loss, and treatment costs per cart owner, per EL affected cart owner, and per cart animal are presented in Tables 1–**5**.

**Table 1:** Annual economic loss due to mortality per cart owner and per affected cart owner by town.

**Town**	**No died**	**Average economic loss/cart owner (ETB)**	**Average economic loss/affected cart owner (ETB)**
Gondar Town	137	4382.11	4824.75
Woreta Town	11	4233.33	8466.70
Overall	148	4364.11	5081.22

*ETB, Ethiopian birr*.

#### Mortality Loss

The annual average economic loss due to mortality of horses or mules per cart owner was estimated to be 4364.11 ETB. When only EL affected cart owners were considered, the annual average economic loss due to mortality was ETB 5081.22 per affected cart owner (Table 1).

#### Working Power Loss

The annual average economic loss due to working power loss attributed to EL per cart owner was estimated to be ETB 2837.98. High working power losses were recorded in Gondar town in which it was estimated to be ETB 3039.11, while in Woreta town it was ETB 1376.42. When only affected cart owners were considered, the annual average economic loss due to working power loss was ETB 3304.32 (Table 2).

**Table 2:** Annual economic loss due to working power loss per cart owner and per affected cart owner by town.

**Town**	**Average days of illness**	**^†^Average effective working days lost**	**Average income from cart animal/day (ETB)**	**Average economic loss/cart owner (ETB)**	**Average economic loss/affected cart owner (ETB)**
Gondar Town	14.78	12.26	232.34	3039.11	3346.10
Woreta Town	8.67	7.19	166.67	1376.42	2752.83
Overall	14.04	11.65	224.40	2837.98	3304.32

† *An adjustment factor of 0.83 (305/365) was used to change the days of duration of illness to actual effective working days lost. ETB, Ethiopian birr*.

#### Treatment Costs

The treatment cost was estimated from medication costs and extra labor costs for caring and seeking treatment for sick animals. The average annual economic loss related to EL treatment costs per cart owner was estimated to be ETB 1244.94. Town wise, Gondar town had the larger treatment costs than Woreta town. When only EL affected cart owners were considered, the annual average economic loss due to treatment costs was ETB 1449.51 (Table 3).

**Table 3:** Annual economic loss due to treatment costs per cart owner and per affected cart owner by town.

**Town**	**Average treatment expenditure/head (ETB)**	**Average cost of extra labor for seeking treatment for sick animals (ETB)**	**Average economic loss/cart owner (ETB)**	**Average economic loss/affected cart owner (ETB)**
Gondar Town	1184.74	116.80	1394.78	1535.66
Woreta Town	107.17	42.11	156.11	312.22
Overall	1054.39	107.77	1244.94	1449.51

*ETB, Ethiopian birr*.

#### Overall Average Economic Losses

The annual overall average economic losses associated with EL per cart owner were estimated to be ETB 8447.03. The largest component (52%) of the economic losses was due to mortality, while treatment costs were the least contributor (15%) to the overall economic losses. When only EL affected cart owners were considered, the annual overall average economic losses were ETB 9835.04 per affected cart owner (Table 4). The difference in overall average economic losses per cart owner between Gondar town and Woreta town was statistically significant (*P* = 0.0002).

**Table 4:** The overall average economic losses of epizootic lymphangitis per cart owner and per affected cart owner per year by town.

**Town**	**Losses due to working power loss (ETB)**	**Treatment costs (ETB)**	**Mortality loss (ETB)**	**Overall average economic loss/cart owner (ETB)**	**Overall average economic loss/affected cart owner (ETB)**
Gondar Town	3039.11	1394.78	4382.11	8,816	9706.51
Woreta Town	1376.42	156.11	4233.33	5765.86	11531.73
Overall	2837.98	1244.94	4364.11	8447.03	9835.04

*ETB, Ethiopian birr*.

The average economic losses associated with EL per cart animal per year were estimated to be ETB 3403.46, 2213.27, and 970.90 for mortality loss, working power loss, and treatment costs, respectively. Gondar town shares the largest overall average economic losses (6719.89) per cart animal level as compared to Woreta town (5405.49) (Table 5).

**Table 5:** Animal level average economic losses of epizootic lymphangitis by town.

**Economic losses (in ETB)**	**Town**		**Overall average economic loss/cart animal (ETB)**
	**Gondar Town**	**Woreta Town**	
Mortality losses per cart animal	3340.21	3968.75	3403.46
Loss due to working power reduction per cart animal	2316.53	1290.39	2213.27
Treatment costs per cart animal	1063.15	146.35	970.90
Overall losses per cart animal	6719.89	5405.49	6587.62

*ETB, Ethiopian birr*.

### Cart Animal Owners' Knowledge and Practices Related to Epizootic Lymphangitis

From the total of the 274 respondents that were recruited for the study, only 90.5% (248/274) were aware of the disease and able to provide responses for knowledge and practice related questions. Most of these respondents expressed that they had experienced the disease in their animal and further indicated that the disease occurs mostly from months August–November. The whole knowledge and practice analysis was done based on these 248 respondents.

#### Socio-Demographic Characteristics

Respondents' mean household size was 4 (range, 1–10). Of all respondents, 98.0% (243/248) were males. Respondents' mean age was 36.1 (range, 19–84) years old. About half of the respondents had primary school education; 25.4% had high school or above educational level, and 24.6% of respondents were illiterate. Most respondents used cart animals (cart business) as a primary source of income; only 7.7% of the respondents were using carts as secondary source of income.

#### Scores on Individual Knowledge and Practice Questions

Most of the knowledge questions were scored high. But three knowledge questions, namely, knowledge about predisposing factors, timing of treatment, and prognosis of the disease, were scored low, with <50% of total obtainable score indicating knowledge gaps in these aspects of the diseases. Relatively low scores were recorded for practice questions in which most of the questions were scored <50%. The practice questions such as avoiding buying new cart animals from EL infected areas, avoiding mingling of healthy and infected cart animals, and taking early cases of the disease for treatment were scored very low (Table 6).

**Table 6:** Individual knowledge and practices questions' scores.

**I**	**Knowledge questions**	**Scores (%)**
1	Do you know the disease EL (Nidift)? A) Yes (1) B) No (0)	248 (100)
2	EL affects which species of animals? A) equines (1) B) Include other animals (0)	201 (81.0))
3	What are the most susceptible species to EL? A) horse (1) B) Other equines (0)	225 (91.7)
4	The most important clinical signs (Out of 3, with 1 point for each choice): A) freely movable cutaneous nodules in legs, chest wall, and the neck. B) Nodules appearance follows lymphatic line and cord like thickening of lymphatics C) chronic, debilitating, pyogranulomatous, and severe wound	564 (75.8)
5	Is EL transmissible between animals? Yes (1), No (0)	224 (90.3)
6	What are the modes of transmission of EL (Out of 3, with 1 point for each choice)? A) contact B) vehicle (harness, whip, brush) C) fly and tick bits	402 (54.0)
7	Clinical course of the disease: A) less than 1 month (0) B) greater than 1 month (1)	248 (100)
8	Do you think is EL a curable disease? A)Yes (1) B) No (0)	117 (47.1)
9	Is the prognosis of the disease good if treated early? A) yes (1) B) No, it doesn't make a difference (0)	119 (47.9)
10	What are the risk factors (Out of 4, with 1 point for each choice)? A) Pre-existing wounds B) Share of harness, whips, cleaning brushes C) Gathering with other infected cart animal D) Housing/feeding together with infected animals	311 (41.8)
**II**	**Practice questions**	
1	Did you get any training on EL before this time? Yes (1), No (0)	109 (44.0))
2	Do you seriously check horses and mules for EL when buying new horses or mules? Yes (1), No (0)	220 (88.7)
3	Do you avoid buying horses/mules from a known infected area? Yes (1), No (0)	54 ((21.7)
4	Do you separate infected from uninfected cart animal at home (i.e., housing, feeding/watering separation)? Yes (1), No (0)	105 (42.3)
5	Do you avoid mingling with affected horse at grazing or at work? Yes (1), No (0)	61 (24.6)
6	Do you avoid harness damage to prevent EL? Yes (1), No (0)	127 (51.2)
7	Do avoid sharing of harness with infected horse and mule (use of harness that has been used for infected mule or horse)? Yes (1), No (0)	119 (48.0)
8	Do you give a break for infected cart animal to recover? Yes (1), No (0)	198 (39.5)
9	Do you give special food (better care) for infected cart animal? Yes (1), No (0)	49 (19.6)
10	Do you take any measures/traditional medicines to prevent or control EL in cart horse/mule? Yes (1), No (0)	142 (57.3)
11	Do you take sick cart animals for treatment in the early stage of the disease (when 1–2 nodules appear)? Yes (1), No (0)	85 (34.3)

#### Knowledge Level of the Respondents About the Disease

The knowledge assessment result revealed that 51.2% (127/248) of the respondents were found to have a good knowledge level (with greater or equal to mean knowledge score) of the disease. The association of knowledge level of the respondents with socio-demographic factors is presented in Table 7.

**Table 7:** Multivariable logistic regression analysis for the association between socio- demographic factors and knowledge level of epizootic lymphangitis.

**Socio-demographic factors**	**Frequency of respondents**	**Percentage with good level of knowledge**	**Odds ratio (95% CI)**	***P*-value**
**Town**				
Gondar Town	218	87.90	1.00	
Woreta Town	30	12.10	0.054 (0.01, 0.24)	0.000
**Age**				
18–29	66	26.61	1.00	
30–39	99	39.92	0.68 (0.34, 1.36)	0.279
≥40	83	33.47	0.6 (0.26, 1.35)	0.217
**Educational level**				
Illiterate	61	24.60	1.00	
Primary school	124	50.00	1.39 (0.65, 2.98)	0.390
High school and above	63	25.40	2.94 (1.35, 6.42)	0.007

The final model that was fitted through backward elimination of non-significant variables contains the study town and education level as significant predictors of good knowledge. In Woreta town, knowledge level of respondents was only 5% of that of Gondar town respondents and having a high school and above educational level increases knowledge level of the respondents almost by three times than illiterates.

#### Epizootic Lymphangitis Practices

The EL practice assessment result revealed that only 45.2% (112/248) of the respondents had a good practice level in relation to prevention and treatment of EL. The association of practice level of the respondents with socio-demographic factors and knowledge level analyzed using multivariable logistic regression is presented in Table 8.

**Table 8:** Factors influencing practice level of the respondents related to epizootic lymphangitis.

**Socio-demographic factors**	**Frequency**	**Percentage**	**Odds ratio (95% CI)**	***P*-value**
**Town**				
Gondar Town	218	87.90	1.00	
Woreta Town	30	12.10	0.16 (0.03, 0.72)	0.018
**Age**				
18–29	66	26.61	1.00	
30–39	99	39.92	0.89 (0.42, 1.87)	0.760
≥40	83	33.47	0.52 (0.21, 1.25)	0.144
**Educational level**				
Illiterate	61	24.60	1.00	
Primary school	124	50.00	1.29 (0.55, 3.02)	0.559
High school and above	63	25.40	1.27 (0.47, 3.45)	0.639
**Knowledge level**				
Poor	121	48.79	1.00	
Good	127	51.21	6.86 (3.72, 12.64)	0.000

The factors associated with practice level in the final fitted logistic regression model were respondent's town of origin and knowledge level (Table 9).

**Table 9:** The final fitted model for the association of between sociodemographic factors and practice level of the respondents against epizootic lymphangitis.

**Socio-demographic factors**	**Frequency**	**Percentage**	**Odds ratio (95% CI)**	***P*-value**
**Town**				
Gondar Town	218	87.90	1.00	
Woreta Town	30	12.10	0.17 (0.04, 0.77)	0.022
**Knowledge level**				
Poor	121	48.79	1.00	
Good	127	51.21	7.27 (3.99, 13.24)	0.000

A statistically significant difference in practices against EL was observed among respondents with different knowledge levels (*P* = 0.000) (Table 9). Those respondents who had a good knowledge had good practice level of 7.27 times that of respondents who had poor knowledge level. In Woreta town, the good practice level of the respondents was only 17% of that of Gondar town respondents.

## Discussion

### Economic Impacts of Epizootic Lymphangitis

The annual average economic losses associated with EL per household (cart owner) in the study towns were estimated at ETB 8447 (USD 316. 7). This is a significant loss for cart animal owners whose livelihood mainly depends on cart business and who are predominately from the lower economic strata. The current estimate was greater than the previous report of Nigatu and Abebaw (4) in central Ethiopia, where they estimated average economic loss per household per year of ETB 779.1 (52.8 USD). This variation is partly due to inflation. The price of horses and the daily income per healthy cart animal increased by about four-fold (from 1616 ETB to 6778 ETB for horse price, and from 55.3 ETB to 224.4 ETB for daily income) in the time between these two studies. Aside from this inflation, the economic impact of the disease is still higher in the current study area. Qualitative studies in other parts of the country have also indicated that EL is ranked among the most economically important health problems of cart horses (5, 6).

The present study indicated that the maximum economic loss per household level was due to mortality, accounting for 52% of the overall annual economic losses. Working power loss was the second most important economic loss of EL, accounting for 33.6% of the overall annual economic loss. It was observed that the average income gained per head per day for EL affected horse reduced by 67% from the average income gained from healthy cart animals. Scantlebury et al. (6) reported that there was 50% reduction in daily earnings per individual cart animal owner due to decreased working capacity, clients reluctant to use diseased horse, and unproductive feed expense in central Ethiopia. A significant difference in the capacity for working hours and reduction in daily income (ETB 40) between EL-infected and non-infected cart animal was also reported by Bekele et al. (5).

The third major component of the overall annual economic loss of EL was captured from the treatment expenditures and extra labor costs of EL management. The average treatment expenditure/head of ETB 1054.39 estimated in the current study was higher than a previous report of Worku et al. (24) who estimated ETB 579. This higher estimate may be attributed to inclusion of the extra labor cost spent for management of EL and price inflation of drugs in the current study. The treatment cost for most cart owners in Ethiopia is expensive, and its efficacy is not reliable especially if the treatment is started late in the course of the disease (25, 26). The effort of mitigating the disease problems should therefore focus more on the development of vaccines that are affordable and could prevent the disease in the first place and could have a potential to eliminate the disease.

### Owners' Knowledge and Practice Related to Epizootic Lymphangitis

The present study aimed to assess the knowledge and practices of cart owners and highlighted key factors affecting EL knowledge and practices in the study area that could be targeted to improve knowledge and practices and thereby help prevention and control of the disease.

Most of the respondents (90.5%) in the study towns were able to correctly describe clinical pictures of EL, and they locally called the disease “Nidift.” This finding agrees with reports generated from focus group discussions of the impact of EL in Ethiopia by Scantlebury et al. (6), in which above half of the participants were able to explain the disease. Another study that assessed owners' knowledge on working equids disease in Ethiopia ranked EL as number 1 and indicated a considerable impact on working ability (27). Most of the respondents in the present study expressed that they had experienced the disease in their animal and further indicated that the disease occurs mostly from months August–November, which could be associated with an increase of the fly population during this end of rainy season. This knowledge of the cart owners is consistent with reports by Endebu and Roger (28) and Ameni (2) indicating seasonal occurrence of the disease.

The knowledge assessment results showed that approximately 52% of respondents were classified as having good knowledge level of the disease EL. Though simple majority of the cart owners have good knowledge about the disease, still it is low compared to the proportion who know the disease by name. This was particularly true for knowledge questions related to predisposing factors of the disease and timing of treatment, both of which have crucial influence on prevention and control of the disease. This calls for more education of the cart owners about the disease, as the knowledge of the nature of the disease is the preliminary step to take an action toward hygienic and managerial measures for prevention and control of the disease.

The knowledge level of the respondents in Woreta town was only 5% of knowledge level of that of Gondar town respondents. These might be due to the frequent trainings given about the disease and other welfare issues of cart horses by University of Gondar as part of its community service activity in Gondar town. Scantlebury et al. (6) reported that cart horse owners who have been exposed to different training on welfare of cart animals by Society for Protection of Animals Abroad (SPANA) had a better knowledge and practice level than cart horse owners who had not attended those trainings in similar communities. Statistically significant knowledge level variation was observed in the cart animal owners that were at different educational levels. Respondents that were at high school and above educational level were around three times better in explaining EL than those without any formal education highlighting the importance of literacy for better animal husbandry knowledge. Similar studies for other diseases also documented better level of knowledge in educated than non-educated animal owners (29, 30).

In this study, approximately 55% of the respondents were at poor practice level related to the disease and respondents of Woreta town had only 17% of good practice level of that of Gondar town respondents. Although the majority (52%) of respondents had good level of knowledge, the proportion of respondents with good practice level was lower (45%), which indicates that knowledge may not be directly translated into practice. Some cart owners explained that there is a practical difficulty in implementing some prevention measures such as isolating infected cart animals both at home and at cart stations, and financial problems to change the previously used harness of infected animals. But still, a statistically significant (*P* < 0.05) association between the practice level and the knowledge level of the respondents on EL was observed. Those respondents who had a good knowledge were 7.27 times likely to have good level of practice than respondents who had poor knowledge level. This is in line with the finding reported by Scantlebury et al. (6), in which cart owners who had a direct exposure to SPANA trainings were better in both knowledge and practices toward EL management and hygienic measures to prevent the disease. A positive relationship between knowledge and practice of animal owners was reported by other researchers for other diseases (29, 31, 32). This indicates that there is a room for improvement of animal owners' good practices related to EL by increasing the level of knowledge of animal owners through knowledge transfer intervention, which was found to be effective in increasing knowledge of working equids owners (33, 34).

The data used for the analyses were based on cart animal owners' diagnosis of the disease. Although EL is relatively easily identifiable disease, the accuracy of owners' diagnosis could have limitation. Moreover, the data were generated based on 1 year period of owners' recall, which inevitability might introduce some degree of recall bias.

## Conclusions

The present study results indicated that EL is causing considerable economic losses on cart animal owners in the study areas who mainly depend on cart business for their living. Concerned bodies should give attention for the control of this disease, which is a threat for the livelihood of poor cart owners and welfare of the cart animals. Cart animal owners in the study areas have a good level of knowledge relative to their level of good practice related to the disease. Socio-demographic factors such as owners' educational level and place of residence were found significantly associated with the good knowledge level of the disease, and knowledge level of the respondents was in turn significantly positively associated with practice level of the respondents. This indicates that extension work that improves awareness and knowledge of the disease can have a positive impact on good practice of owners in prevention and control of the disease, and therefore should be given attention to mitigate the impact of the disease on the welfare of cart owning community and the cart animals.

## Data Availability Statement

The raw data supporting the conclusions of this article will be made available by the authors, without undue reservation.

## Ethics Statement

The studies involving human participants were reviewed and approved by Institutional Board of University of Gondar. Written informed consent for participation was not required for this study in accordance with the national legislation and the institutional requirements.

## Author Contributions

AM designed the study, collected the data, analyzed the data, and drafted the manuscript. TF conceived the study, designed the study, and reviewed the manuscript. WJ conceived the study, designed the study, analyzed the data, and revised the manuscript. All authors contributed to the article and approved the submitted version.

## Conflict of Interest

The authors declare that the research was conducted in the absence of any commercial or financial relationships that could be construed as a potential conflict of interest.
