# Supplementary material for: Estimating the Economic Impact and Assessing Owners' Knowledge and Practices of Epizootic Lymphangitis in Equine Cart Animals in Central and South Gondar Zones, Amhara Region, Ethiopia
Source: Front Vet Sci. 2021 Jun 16;8:673442. doi: 10.3389/fvets.2021.673442 (PMC8245057; doi:10.3389/fvets.2021.673442)
Supplement: Supplementary file 1 [file Data_Sheet_1.pdf]

The supplementary material

Supplementary material 1: **Questionnaire on economic impact of epizootic lymphangitis, and knowledge and practice related to epizootic lymphangitis.**

I. General information

Owners' name (optional): ____
Address**:**
Zone___________________Woreda____________Kebele____________Tel_________.
SEX: □ Male □ Female; Age: _____, # of family ______, Primary source of Income_____.

Educational Level: □ Illiterate □ primary (read and write) □ High school complete and above.

1**.** Do you know the disease epizootic lymphangitis (*nidift* in vernacular name)?

□ Yes □ no

2**.** If yes to above, can you describe the clinical signs associated with this disease? (*If he /she mentioned one or more of the following typical symptoms or epidemiological features of the disease, then the person will be deemed to know the disease and continue with the questionnaire*).

A. Freely movable cutaneous nodules developed under the skin and follow a lymphatic line and transmissible.

B. Nodules mostly seen in legs, chest wall and neck and make line, contagious and chronic.

C. Ruptured nodule and discharge thick yellow pus, ulcerating and spreading and make a line.

D. lymphadenopathy, lymphangitis and cord like thickening of lymphatics and neighboring glands

II. Economic impact questions

Would you please provide the following information about the morbidly and mortality of epizootic lymphangitis in your cart animals in the last one year?

1- Number of cart animal owned: horse ___________ Mule ____________.
2- Number of horse and mule died by EL in the last one year? ______________________.
3- Number of horse and mule affected by the disease in the last one year? _____________.
4- *For each affected animal*
   a. Number of animals treated _____________________, average treatment cost/head if there is any: _____________________________________birr.

b. Fate of the animals: A) died (abandoned) B) recovered C) still sick/diseased
c. If died, the estimated value/price of died animal: _____________ birr.
d. If recovered/still diseased, how long was the illness period? ________________Days.
e. If recovered and/still diseased, how much percentage reduction was there in the working power of the animal (working time/day)? _____________________________________.
f. What is the average daily income gained from a normal car horse/mule per day? ___________________________.
g. If the animal is still sick what is the chance of recovery? ________________. Estimated value/price of the animal ____________. And time duration of illness without work________________.
h. ***Cost related to care infected animal:***
   - Average number of working hours lost for managing the sick animals. ____________.
   - Average number of working hours lost for seeking treatment for sick animals______.
   - Average payment rate of a replacement laborer/hour in the locality of herd? ____birr
   - Cost of additional feed supplementation for the diseased one if any?

III. Knowledge and Practice Questions

a. Knowledge questions

1. Do you know the disease EL (Nidift)? A. Yes (1) B. No (0)
2. EL affects which species of animals? A. equines (1) B. Include other animals (0)
3. What are the most susceptible species to EL? A. horse (1) B. Other equines (0)
4. The most important clinical signs (Out of 3, with 1 point for each choice): A) freely movable cutaneous nodules in legs, chest wall and the neck. B) Nodules appearance follows lymphatic line and cord like thickening of lymphatics C) chronic, debilitating, pyogranulomatous and severe wound
5. Is EL transmissible between animals? Yes (1), No (0)
6. What are modes of transmission of EL (Out of 3, with 1 point for each choice)? A) contact B) vehicle (harness, whip, brush) C) fly and tick bits
7. Clinical course of the disease: A) less than one month (0) B) greater than one month (1)
8. Do you think is EL a curable disease? A) Yes (1) B) No (0)
9. Is the prognosis of the disease good if treated early? A. yes (1) B. No, it doesn't make a difference (0)
10. What are the risk factors (Out of 4, with 1 point for each choice)? A) Pre-existing wounds B) Share of harness, whips, cleaning brushes C) Gathering with other infected cart animal D) Housing/feeding together with infected animals

b. Practice questions

1. Did you get any training on EL before this time? Yes (1), No (0)
2. Do you seriously check horses and mules for EL when buying new horses or mules? Yes (1), No (0)
3. Do you avoid buying horses/mules from a known infected area? Yes (1), No (0)
4. Do you separate infected from uninfected cart animal at home (i.e., housing, feeding/watering separation)? Yes (1), No (0)

5. Do you avoid mingling with affected horse at grazing or at work? Yes (1), No (0)
6. Do you avoid harness damage to prevent EL? Yes (1), No (0)
7. Do avoid sharing of harness with infected horse and mule (use of harness that has been used for infected mule or horse)? Yes (1), No (0)
8. Do you give a break for infected cart animal to recover? Yes (1), No (0)
9. Do you give special food (better care) for infected cart animal? Yes (1), No (0)
10. Do you take any measures/ traditional medicines to prevent or control ELcart horse/mule? Yes (1), No (0)
11. Do you take sick cart animals for treatment in the early stage of the disease (when 1-2 nodules appear)? Yes (1), No (0)
